# Hotel Dieu

**Published:** 1829-08

**Authors:** 


### HOTEL DIEU.

### *Poisoning by Acetate of Copper. Efficacy of Albumen.*

A MAN, aged twenty-six, of fair complexion, and not very robust, who had lived in Paris for the last four years, had experienced several attacks of vomiting and purging, was thin, pale, and of a very melancholy character. In 1824, he tried to poison himself by eating some cicuta virosa, but from the effects of which he soon recovered.

On the 18th October, 1825, he put eight sous pieces into a glassful of strong vinegar, and suffered them to remain there until the 25th. On that day he made a full dinner at two o'clock in the afternoon, and drank a bottle of red wine. At four o'clock he took half a glassful of the vinegar and copper, taking care to stir the money previously. After the lapse of another quarter of an hour, he drank the remainder of this mixture: he then washed the sous afresh with a little vinegar, and afterwards with a small quantity of brandy, and drank the whole. At seven o'clock his comrades found him stretched without sense upon the floor, and brought him immediately to the Hôtel Dieu.

On his admission, it was observed that all the muscles were agitated with violent convulsions, the limbs remaining rigid in the intervals. There was much difficulty in supporting the patient. The teeth were firmly closed; the breathing short; the pulse hard, small, and very slow. The stomach was tender upon pressure, which produced violent convulsions. No intelligence could be obtained as to the substance swallowed: the lips did not retain any relics that indicated its nature, nor had the breath any particular odour. The mouth was opened at length, and some glasses of warm water forced down; the uvula and pharynx were tickled with a feather, but without effect. He drank in the interval between the convulsions; and in about a quarter of an hour he came to his recollection in some degree, and explained the cause of his symptoms. The whites of eggs were then mixed, and beaten up with water, and he swallowed immediately several large glasses. The convulsions ceased immediately, but the hiccup continued during part of the night.

On the 26th, in the morning, the pulse was large, slow, and intermitting; the belly contracted, hard, and very sensible all over to the slightest pressure; slight convulsions in the limbs; general lowness, taciturnity, and paleness; the pupils were dilated; the tongue soft, moist, and pale. Thirty leeches were applied to the abdomen. Emollient cataplasms, decoction of linseed, clysters, and a rigid diet, were prescribed. In the evening, the patient was worse; the agitation was extreme, attended with colics, dyspnœa, hiccup, and a hard and contracted pulse. Forty leeches were applied to the belly. The urine was scanty and scalding. The use of the water, with albumen, was continued. One hard motion followed the third clyster. The night was passed badly.

27th.—The amendment was striking: the pulse had become soft, the abdomen free from pain, the urine free, and a liquid motion had passed. The same treatment was continued.

From this day the amendment continued; and in ten days the digestive functions were re-established, and all the bad symptoms had disappeared. The moral condition of the man had not, however, improved: he still continued taciturn, motionless, with a pale countenance and a dry hot skin, and slept but little. He quitted the hospital on the 8th of November.

According to the experiments of M. Drousait, as recorded in M. Orfila's work, the acetate of copper is endowed with properties much more active than common verdigris, which is explained by the much greater solubility of the former substance.

In the above case, the substance had been swallowed above three hours; the nature of the poison was then unknown, and there was nothing to lead to that knowledge. It was not known whether the patient had vomited or not; it therefore became necessary to provoke that action of the stomach. The patient, however, recovered in some degree, so as to explain the nature of the poison: then albumen was administered. All the pretended antidotes of copper are not to be compared to this simple means, so easily to be met with every where, and under all circumstances. The sulphurets of potash, of lime and iron, mentioned by Navier, decompose the salt of copper, but the precipitate retains sufficient poisonous properties to produce mischief. Saline and earthy alkalis cause the same effects. The infusion of galls, mentioned by M. Chansarel, is nearly inefficacious. Sugar has been eulogised by M. Marcelen Duval as the real antidote to copper, but the experiments of MM. Orfila and Vogel have proved the contrary. It is not so with albumen: this substance decomposes the salts of copper at an ordinary temperature; it should therefore be immediately administered, favoring the vomiting and purging by proper remedies.—*La Clinique.*

